# Dr. Adams, on Cutaneous Eruptions

**Published:** 1804-09-01

**Authors:** Joseph Adams

**Affiliations:** Worcester


### Medical and Phyfical Journal.

VOL. XII.] SEPTEMBER 1, 1804. [NO. LXVII.

*Printed for R. PHILLIPS, by W. Thorne, Red Lion Court, Fleet Street, London.*

### *To the Editors of the Medical and Physical Journal.*

GENTLEMEN,

THE little leisure I have had since my return from Madeira, has prevented my attending accurately even to the periodical publications of the day. But the name of JENNER, at the close of the first paper in your last Number, could not but arrest my attention, in a journey I am now making in quest of materials for a more correct account of morbid poisons.

Among the subjects which will form a part of my next edition, is one very much connected with Dr. Jenner's paper; and if I mistake not, the particular road of my enquiry has conducted me through some intricacies which have escaped the general practitioner. If Dr. Jenner has remarked them, his numerous engagements have prevented his dilating upon them, in a manner sufficiently explicit for those who have not followed that chain of reasoning which connects every nosological enquiry. It is indeed hardly possible, in the small space allotted to each writer in a production like yours, to offer any thing more than an accurate statement of facts; and perhaps you will charge me with obscurity in attempting to compress a complicated enquiry into so small a compass. However, as Dr. Jenner expresses an intention to publish more at large, I shall be glad to be confirmed or corrected by him.

When the constitution is engaged in any particular action, which produces local affection, any cause which at another time would produce an uniform effect, is more or less influenced by this state of the system. This is more remarkable in morbid poisons than in other diseases. The law of small-pox is to produce slough. Let a blister be applied in the early stage of the eruption, and if the disease is violent, a thin slough will be formed of the same extent as the vesication. If, whilst the constitution is under the influence of erysipelas, a venereal chancre is contracted, the whole prepuce or glans will be suffused with an erysipelatous efflorescence; and as the erysipelas either yields to art, or subsides of itself, the chancre acquires its true character.

This subject might be extended much further, but my only wish is to render myself intelligible. If then the constitution thus engaged, should alter the usual appearance excited by local stimuli, we should not wonder if, on some occasions, she should be altogether insensible to those stimuli which would affect her at other times. But a disease may continue so long as to be almost familiar with the constitution; that is, the local effects may remain after the constitutional action which formed them has subsided; or the constitutional action may be so weak as to be easily superseded by a new and more powerful stimulus. In this case the insertion of the vaccine, or any other morbid poison, will not only produce its genuine effect, but the constitution will be so engaged by it, that inflammation at a distance from the insertion, and excited by any previous cause, will now partake of this new action, and perhaps be altogether superceded by it; and if the new action is of such a nature as to subside after passing through a determined course, the patient will by these means be relieved of his inveterate disease whilst he is passing through the new one.

These considerations are of the utmost importance, because some of the honest but over sanguine friends of vaccination have too generally recommended the operation as a means of clearing the skin from other eruptions. It is with particular pleasure I see Dr. Jenner's cautions on the subject, and also *his* remark that the vaccine pustule or vesicle is not always imperfect or ineffective during the influence of a malady which *he* describes; on the contrary, he observes, that it is sometimes perfectly correct, and much more frequently so when the first disease has been of long standing, than when in its recent state; and what is remarkable, the first disease is then sometimes entirely swept away." *

There is a kind of itch to which the poorer people of Madeira are extremely liable, and which I shall hereafter describe more at large. When such a patient is inoculated with

* See the paper before alluded to.

with vaccine lymph, for the most part no local effect appears. I have repeated the inoculation in some subjects for three times with recent matter, but with as little success. In one of these, after she was cured of the first disease, I was astonished at the sight of a large number of vesicles in various parts of the body. This was not the only instance in which I had reason to believe, that though the local effect of vaccination might be superceded by other causes, yet when those causes ceased, a disposition to the disease, which was formed at the time of insertion, is now brought into action, and shows itself by a general eruption. I very much think that this occurrence is more general than is suspected, and that some of those eruptions which have appeared at a remote period after vaccination, and which the zealots on one side have called small-pox, and those on the other side chicken-pox, have been vaccine vesicles. I have already given cases of this, arising from a peculiar state of the atmosphere; and I cannot help wishing that we were all more attentive to mark phænomena, than hastily to get through difficulties.

The cow-pox stands so deservedly high in the estimation of all reasoning people, that its friends ought as much as possible to encourage the objections of the captious, in order to ascertain every variety, and reduce them to laws. The objections hitherto started are too light to be taken into account when compared with the contrary evidence. But they ought all to be circumstantially detailed, candidly examined, and carefully compared, so as to enable us to reduce these anomalous appearances, as they are called, to fixed laws. That this is possible I have no doubt; and while we are forming associations for diffusing this invaluable blessing, would it not be desirable to have a centre of communication to which every anomalous case should be referred. Let a medical committee be formed for this purpose. This appears to me the more necessary, because it has been too much the fashion, either publickly or privately, to address the discoverer of the invention on all these occasions. It is equally cruel and absurd to suppose him aware of every little variety that may occur in a practice, the value of which he had no sooner ascertained than he generously imparted it to the public. Is it thus we show our gratitude for the greatest improvement the art has ever received, by perpetually persecuting the generous inventor? If such a committee were formed, every fact would have its due importance affixed to it, and the public mind would not be continually harrassed by crude and

 contradictory contradictory reports. This committee should be formed in the metropolis, and allowed to receive and send their letters and packets from all parts of the world, free of an expence, which I conceive at present must swallow up the whole interest of the pittance voted by the representatives of the richest nation in the world. But it becomes me to be cautious, lest I should touch on topics which may have already been discussed during my absence from England; I therefore leave the subject to the consideration of your better informed Correspondents.

*Worcester,*
*August* 9, 1804.

I am, &c.

JOSEPH ADAMS.

